# Positioning centrioles and centrosomes

**DOI:** 10.1083/jcb.202311140

**Published:** 2024-03-21

**Authors:** Matthew R. Hannaford, Nasser M. Rusan

**Affiliations:** 1 Cell and Developmental Biology Center, National Heart, Lung, and Blood Institute, National Institutes of Health, Bethesda, MD, USA

## Abstract

Centrosomes are the primary microtubule organizer in eukaryotic cells. In addition to shaping the intracellular microtubule network and the mitotic spindle, centrosomes are responsible for positioning cilia and flagella. To fulfill these diverse functions, centrosomes must be properly located within cells, which requires that they undergo intracellular transport. Importantly, centrosome mispositioning has been linked to ciliopathies, cancer, and infertility. The mechanisms by which centrosomes migrate are diverse and context dependent. In many cells, centrosomes move via indirect motor transport, whereby centrosomal microtubules engage anchored motor proteins that exert forces on those microtubules, resulting in centrosome movement. However, in some cases, centrosomes move via direct motor transport, whereby the centrosome or centriole functions as cargo that directly binds molecular motors which then walk on stationary microtubules. In this review, we summarize the mechanisms of centrosome motility and the consequences of centrosome mispositioning and identify key questions that remain to be addressed.

## Teaser

Hannaford and Rusan summarize the direct and indirect transport mechanisms by which centrosomes and centrioles are positioned in cells.

## Introduction

The centrosome is a membraneless organelle that functions as the main organizer of the microtubule cytoskeleton throughout eukaryotes (Azimzadeh and Bornens, 2007; Bornens, 2021). Centrosomes comprise two barrel-shaped structures called centrioles surrounded by a proteinaceous matrix of proteins called the pericentriolar material (PCM). The PCM provides the platform required for nucleating and anchoring microtubules (Box 1) (Woodruff et al., 2014; Pimenta-Marques and Bettencourt-Dias, 2020).

Box 1 Definitions

**Figure 1.**
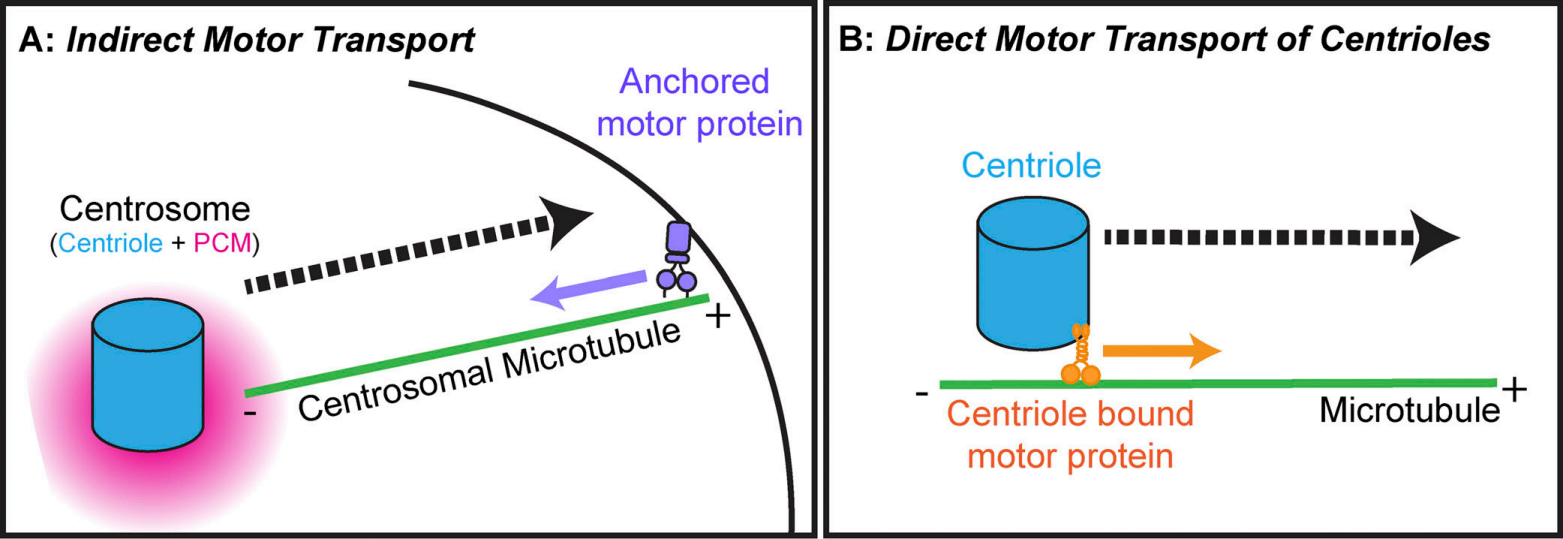
**Indirect versus direct motor transport. (A)** Indirect motor transport occurs when a microtubule (green) nucleated and anchored at the centrosome is engaged by a motor protein, for example, Dynein (purple) at the cell cortex. These motor proteins will exert force on the microtubule thereby moving the centrosome. +/− indicates microtubule polarity. **(B)** Direct motor transport occurs when motor proteins (orange) anchored to the centriole (blue) engage microtubules (green) and pull the centriole directly along the microtubule as cargo, in this example, Kinesin-1 is moving the centriole toward the microtubule-plus end.

The centriole is a cylindrical organelle made of a ninefold symmetric ring of microtubule triplets. Centrioles act as a platform for the recruitment of PCM, which is a matrix of proteins that recruit the gamma-tubulin ring complex, thereby nucleating and anchoring microtubules (Fig. 1 A). The centrosome is the organelle comprised of the centriole and PCM. The microtubules nucleated from the centrosome can be referred to as centrosomal microtubules. When the centrosome is nucleating microtubules, it can be referred to as the microtubule organization center (MTOC).

Early observations of centrosomes in cultured cells described their location near the center of interphase cells from where they radiate a microtubule array. It is this microtubule array that holds the centrosome in the center of the cell through the balance of pushing and pulling forces exerted on the microtubules. Local depolymerization of microtubules with nocodazole demonstrated that microtubule forces were necessary to maintain centrosome positioning (Burakov et al., 2003). This radial interphase microtubule array is stabilized, in part, by the motor protein Dynein; inhibition of Dynein resulted in the loss of force-balance, microtubule buckling, and centrosome mispositioning (Holy et al., 1997; Koonce et al., 1999; Wu et al., 2011). A recent study utilized the targeted severing of specific microtubule populations and the analysis of cytoplasmic flows to determine the precise contribution of Dynein-mediated cytoplasmic pulling forces, cortical pulling forces, and microtubule pushing forces for centrosome centering. These experiments demonstrated that in the *Caenorhabditis elegans* one-cell embryo, cortical Dynein-mediated pulling forces are the primary mechanism of centrosome centering (Wu et al., 2023).

The assumption that centrosomes resided at the center of cells was challenged by the observation that migrating cells decentralize or polarize their centrosome to either the posterior or anterior part of the cell (Luxton and Gundersen, 2011). Careful analysis of polarized cells revealed that centrosome position is influenced by actomyosin-network contractility (Hale et al., 2011). Later studies used adhesive micropatterns to impose polarized actin architecture and revealed that centrosomes moved away from the geometric center of the cell toward the geometric center of the actomyosin network (Jimenez et al., 2021; Yamamoto et al., 2022).

These studies revealed several key players that regulate centrosome movement and positioning, all of which are highly dependent on the centrosome’s capacity to nucleate and anchor microtubules. Changes in actomyosin contractility, actin localization, and microtubule-dependent pulling forces, all alter the geometry of the microtubule network and thereby alter centrosome positioning (Letort et al., 2016). Because these factors are indirectly moving centrosomes by acting on centrosomal microtubules, we termed this transport “Indirect Motor Transport” (Fig. 1 A). However, in some cell types, centrioles lacking PCM, or centrosomes lacking centrosomal microtubules, move along the cytoskeleton as cargo (Hannaford et al., 2022; Li et al., 2017); in these cases, we term the transport as “Direct Motor Transport” (Fig. 1 B). In this review, we describe the diverse mechanisms that mature centrosomes (with PCM and MTOC functionality), and immature centrioles (no PCM, no MTOC function) use to position themselves within cells and the consequences of dysfunctional motility.

### Positioning centrosomes during cell division

An essential goal of cell division is the faithful segregation of chromosomes in mitosis. Failed chromosome segregation results in increased aneuploidy (altered chromosomal copy number), a hallmark of many tumor cell types that is believed to be a driver of malignant transformation (Girish et al., 2023). Correct chromosome segregation requires the mitotic spindle, which is mainly constructed from microtubules generated by centrosomes. It is for this reason that the loss of centrosomes, or the presence of dysfunctional centrosomes, has remarkably negative effects on genome stability (Wang et al., 2020; Farrell et al., 2023).

Not only is the presence of centrosomes vital, but their movement to opposite sides of the nucleus prior to nuclear envelope breakdown (NEBD) is important for proper mitotic spindle formation. Any delay in centrosome separation in prophase results in an increase in merotelic kinetochore attachments (where a single kinetochore attaches to both spindle poles instead of one). These merotelic attachments lead to increased lagging chromosomes and aneuploidy (Silkworth et al., 2012; Cimini, 2023).

Centrosome separation in prophase is a form of indirect motor transport. Faithful assembly of the bipolar mitotic spindle relies on the tight regulation of multiple motor proteins along with the actomyosin cytoskeleton to exert force on centrosomal microtubules to properly position the two centrosomes before and after NEBD (Rosenblatt et al., 2004; Cao et al., 2010). The core motors of the spindle assembly pathway are Kinesin-5 (Eg-5), Kinesin-14 (HSET), and Dynein, all of which have been well-reviewed (Prosser and Pelletier, 2017; Tanenbaum and Medema, 2010; Valdez et al., 2023); we only provide a brief overview.

### The role of Kinesins

Kinesin-5 is a homotetrameric motor protein that localizes to centrosomes and microtubules in prophase upon phosphorylation by Cdk1 (Cahu et al., 2008; Sawin et al., 1992; Sharp et al., 1999a; Blangy et al., 1995). Kinesin-5 bridges antiparallel microtubules and, through its motor activity, forces the microtubules in opposing directions (Kapitein et al., 2005; van den Wildenberg et al., 2008). Kinesin-5 inhibition causes a failure of centrosome separation because of a loss of sliding forces acting on antiparallel centrosomal microtubules emanating from the two centrosomes, ultimately compromising bipolar spindle formation (Heck et al., 1993; Roof et al., 1992; Sawin et al., 1992; Whitehead and Rattner, 1998). This so-called Kinesin-5 pushing model was questioned after experiments demonstrated the independent movement of the two prophase centrosomes (Waters et al., 1993), a result inconsistent with Kinesin-5 pushing the two centrosomes apart. Additional evidence that Kinesin-5 mediated pushing forces were insufficient to explain centrosome separation prior to NEBD came from the inhibition of Kinesin-5 in the syncytial blastoderm of *Drosophila melanogaster*, which did not prevent centrosome separation in prophase. However, Kinesin-5 inhibition did prevent bipolar spindle formation following NEBD (Brust-Mascher et al., 2009), which is consistent with Kinesin-5 functioning to separate spindle poles after Kinesin-5 is released from the nucleus (Sharp et al., 1999a, 1999b). Furthermore, Kinesin-5 inhibitors showed limited success in clinical trials (Box 2) aimed at preventing mitotic spindle formation and thus reducing the proliferative potential of cancer cells (Garcia-Saez and Skoufias, 2021; Shi and Mitchison, 2017). Taken together, these results indicate that other motors likely contribute to centrosome separation in prophase.

Box 2 Modulating centrosome positioning as a pathway to chemotherapy

Microtubules form the fibers of the mitotic spindle that are required for the separation of the chromosomes during mitosis. Due to the elevated proliferation observed in cancer cells, a series of small molecules targeting microtubules still form a widely used category of anticancer agents. Unfortunately, given the critical role of the microtubule cytoskeleton in multiple aspects of cellular physiology, these therapies often induce multiple side-effects including alopecia, neuropathy, and neutropenia. The modes of action and subcategories of MT targeting agents have already been well-reviewed (Čermák et al., 2020; Lafanechère, 2022).

Targeting centrosome separation: In an effort to more specifically target proliferating cells, multiple pharmaceutical companies have developed drugs targeting the normal process of centrosome separating in prophase. The main target of this attempted therapy is Kinesin-5. Although some of these drugs have been deemed safe in early-phase clinical trials, they have exhibited limited efficacy (Shahin and Aljamal, 2022; Tischer and Gergely, 2019). One potential reason for this is that Kinesin-12 can rescue spindle assembly following Kinesin-5 inhibition (Sturgill and Ohi, 2013; Sturgill et al., 2016; Solon et al., 2022).

Targeting centrosome clustering: Another centrosome positioning pathway targeted for cancer therapy is “Centrosome Clustering.” Cancer cells often carry supernumerary centrosomes (Chan, 2011; Marteil et al., 2018), which would normally lead to chromosome missegregation and cell death due to multipolar spindle formation. In some cases, cancer cells overcome this obstacle by hijacking a Kinesin-14-based mechanism that pulls the centrosomes together, causing them to cluster and leading to bipolar spindle formation that escapes terminal multipolar-spindle division (Kwon et al., 2008; Chavali et al., 2016; Vitre et al., 2020). A popular and reasonable hypothesis in the field is that preventing centrosome clustering by inhibiting Kinesin-14 could specifically kill mitotic cancer cells. Thus far, targeting Kinesin-14 s has been effective in preventing centrosome clustering in cultured cells and mouse models, but there is yet to be any clinical trial data (Xiao and Yang, 2016). It is important to note that there is some evidence that, despite its effectiveness in preclinical models, targeting mitosis may not be a clinically effective strategy for treating cancers. It is possible that the effectiveness of microtubule-targeting drugs is due to the critical role microtubules play in the interphase cells that form the bulk of the tumor mass (Komlodi-Pasztor et al., 2011).

A screen for microtubule motors identified Kinesin-12 as a motor cooperating with Kinesin-5 for bipolar spindle assembly (Sturgill et al., 2016; Tanenbaum et al., 2009; Vanneste et al., 2009). Kinesin-12 normally acts on stable Kinetochore (K)-fibers; however, in the absence of Kinesin-5 activity, Kinesin-12 can localize to non-kinetochore microtubules and rescue Kinesin-5 function in centrosome separation, although separation is delayed (Sturgill and Ohi, 2013). Future development of Kinesin-12 inhibitors that are coupled to Kinesin-5 inhibition presents an exciting possible therapeutic strategy (Box 2) (Dumas et al., 2019).

A third Kinesin involved in centrosome positioning for spindle assembly is the minus end–directed motor, Kinesin-14, otherwise known as HSET or ncd (Hatsumi and Endow, 1992a, 1992b; Endow et al., 1994; Endow and Komma, 1996; Sharp et al., 1999b; Fink et al., 2009; Hepperla et al., 2014). Early experiments investigating the function of Kinesin-14 led to the conclusion that it antagonized the pole separating forces of Kinesin-5; Kinesin-14 mutant *Drosophila* embryos injected with Kinesin-5 inhibitory antibodies were capable of forming bipolar spindles, in contrast to Kinesin-5 antibody injected alone (Sharp et al., 1999b). The same mechanism was observed in a human cell line (Mountain et al., 1999). Analysis of cancer cells revealed that Kinesin-14 promotes cancer cell survival by clustering centrosomes at the spindle pole, preventing multipolar spindle formation (Kleylein-Sohn et al., 2012). Therefore, Kinesin-14 has become an important target for potential cancer therapy (Box 2).

### The role of Dynein

Another motor important for centrosome separation is the minus end–directed motor Dynein. Dynein is a large protein complex consisting of two heavy chains, two intermediate chains, two intermediate light chains, and three pairs of light chains. Dynein inhibition in mammalian cells caused monopolar spindles (Vaisberg et al., 1993), similar to the inhibition of Kinesin-5. A comparable result was observed by a time-lapse analysis in the *Drosophila* syncytial blastoderm, which revealed that Dynein mutants failed to fully separate their centrosomes (Robinson et al., 1999).

The regulation of centrosome dynamics by Dynein is complicated by its diverse localizations and functions. During prophase, Dynein localizes to the nuclear envelope and centrosomes, while in prometaphase, Dynein additionally localizes to the cell cortex. The subcellular functions of different Dynein motor populations are difficult to discern experimentally. However, mathematical modeling has enabled the in silico separation of Dynein populations and proposed that cortical Dynein is not essential for spindle formation (Mercadante et al., 2021). One method that has been successfully used to test the role of Dynein subpopulations is to investigate Dynein adaptors. For example, Dynein localizes to the outer nuclear membrane via its adaptor BicD and nuclear pore complexes (Gibson et al., 2022). Knockdown or mutation of BicD led to failed centrosome separation prior to NEBD, thus revealing the specific role of nuclear envelope Dynein (Bolhy et al., 2011; Splinter et al., 2010). The loading of Dynein to the nuclear envelope in prophase depends upon the linker of nucleoskeleton and cytoskeleton complex (LINC complex) (Malone et al., 2003; Minn et al., 2009). In prophase, the centrosomes are positioned opposite each other by the LINC complex at the shortest axis of the nucleus, which facilitates robust spindle assembly (Nunes et al., 2020). Disruption of the LINC complex caused centrosome mispositioning in prophase and delayed spindle assembly (Lima et al., 2024). Interestingly, the LINC complex also interacts with the actomyosin network, which is known to influence centrosome positioning (Nunes et al., 2020; Stiff et al., 2020). It is now important to dissect the precise contribution of the actomyosin network in regulating LINC–Dynein mediated pulling forces at the nuclear envelope and further characterize the interplay between the outer and inner nucleoskeleton.

Following NEBD, Dynein influences centrosome positioning via its localization at the cell cortex in prometaphase. Cortical Dynein pulls on astral microtubules thereby positioning centrosomes and orienting the mitotic spindle. This is a highly conserved spindle orientation process that involves the adaptor proteins LGN and NuMA anchoring Dynein and its binding partner Dynactin to the cortex (Kiyomitsu and Cheeseman, 2012; Okumura et al., 2018; Pirovano et al., 2019). This centrosome positioning and spindle orientation machinery has been identified in several systems including *C. elegans* zygotes, *Drosophila* neuroblasts, and mouse skin/neuroepithelial progenitors (di Pietro et al., 2016).

### Motility of centrosomes and centrioles in interphase cells

In addition to the centrosome movements necessary for spindle formation and orientation, centrioles and centrosomes are motile in several other contexts.

### Centrioles trigger cytokinesis

**Figure 2.**
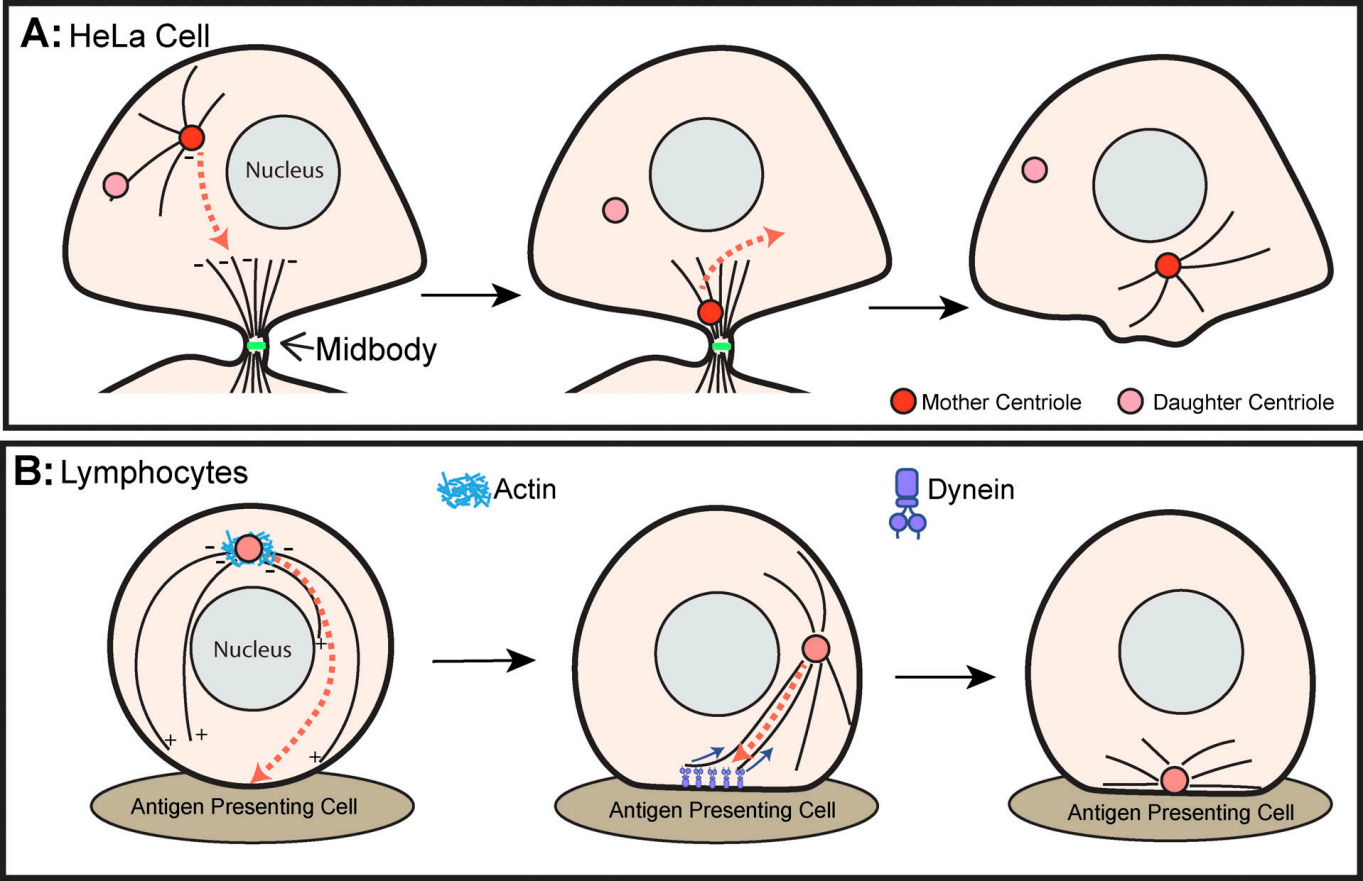
**Centrosome translocation in interphase cells. (A)** During cytokinesis of cultured cancer cells, the mother centriole (red) migrates (arrow) into the cytokinetic furrow along the midbody microtubules. Upon abscission, the centriole exits the cytokinetic furrow. **(B)** Upon interaction between lymphocytes and antigen-presenting cells, centrosomal actin (light blue) disassembles and dynein (purple) clusters at the immune synapse. Microtubule nucleation at the centrosome is increased and these centrosomal microtubules (black) contact the clustered dynein motors, which then pull on the microtubules to move the centrosomes to the center of the immune synapse. +/− indicates microtubule polarity.

Electron micrographs acquired in the 1970s document centrioles proximal to the cytokinetic furrow and midbody at the end of mitosis (Rattner and Phillips, 1973). Later immunofluorescence experiments revealed that the positioning of centrioles to the cytokinetic furrow was coincident with the emergence of the midbody microtubule array (Mack and Rattner, 1993). Live imaging determined that after telophase only the mother centriole becomes highly dynamic, migrating along midbody microtubules toward the site of abscission and then back toward the center of the cell as cytokinesis is completed (Piel et al., 2000) (Fig. 2 A). These results led to the model that the mother centriole delivers an unknown signal that triggers abscission (Piel et al., 2001).

In contrast to this model, other studies have suggested centrioles are not essential for cytokinesis (Hinchcliffe et al., 2001; Khodjakov and Rieder, 2001) and that the movement toward the intracellular bridge is cell-line dependent (Jonsdottir et al., 2010). Therefore, the function and prevalence of midbody-directed migration remain to be uncovered. Nevertheless, careful imaging of centriole dynamics did reveal centriole motility independently of their role as a microtubule organizer (Piel et al., 2000).

### Centrosome positioning at the immune synapse

Lymphocytes (T and B cells) play a key role in the adaptive immune response by binding to infected cells and producing antibodies or directionally secreting cytotoxic vesicles to kill infected cells (Booth and Toapanta, 2021). This requires the formation of an immune synapse between the lymphocyte and an antigen-presenting cell (Douanne and Griffiths, 2021). Following the establishment of the T cell immune synapse, the centrosome undergoes extreme relocalization from above the nucleus to the membrane at the site of the immune synapse (Fig. 2 B). This centrosome position enables the minus end–directed transport of cytotoxic vesicles along centrosomal microtubules, which facilitates secretion at the synapse (Geiger et al., 1982; Stinchcombe et al., 2006). Therefore, centrosome migration to the immune synapse is necessary for efficient immune synapse function (Fig. 2 B). Live-cell imaging experiments revealed that centrosome movement to the immune synapse depends on the motor protein Dynein. Cortical Dynein clusters at the center of the immune synapse where it engages centrosomal microtubules leading to centrosome migration (Combs et al., 2006; Yi et al., 2013; Sanchez et al., 2019; Gros et al., 2021).

Like T cells, centrosomes migrate through the B cell cytoplasm in a Dynein-dependent manner (Reversat et al., 2015). Centrosome movement to the immunological synapse is coincident with an increase in microtubule nucleation from the centrosome, a downregulation of Arp2/3, and a loss of F-actin around the centrosome (Obino et al., 2016). While the exact role of reduced actin around the B cell centrosome is not known, in vitro experiments suggest that the presence of actin filaments could limit the growth of microtubules around the centrosome (Inoue et al., 2019). If this is true in B cells, the reduction in actin might be important for the increase in centrosomal microtubules, thus driving centrosome migration.

Though the mechanism of centrosome positioning to the immune synapse appears well-dissected, multiple questions remain. It remains unknown how Dynein is tethered at the immune synapse and how Dynein-mediated pulling is combined with microtubule shortening, both of which are required to drive centrosome repositioning.

### Centrosome motility for ciliogenesis

Motile cilia and non-motile cilia are microtubule-based organelles found in many eukaryotic cells. Motile cilia function primarily to move fluid through lumens of epithelial tissues, while non-motile cilia are predominantly considered signaling organelles (Focşa et al., 2021). Cilia formation at the cell surface requires that centrosomes migrate to and dock at the cell cortex (Focşa et al., 2021; Reiter and Leroux, 2017).

**Figure 3.**
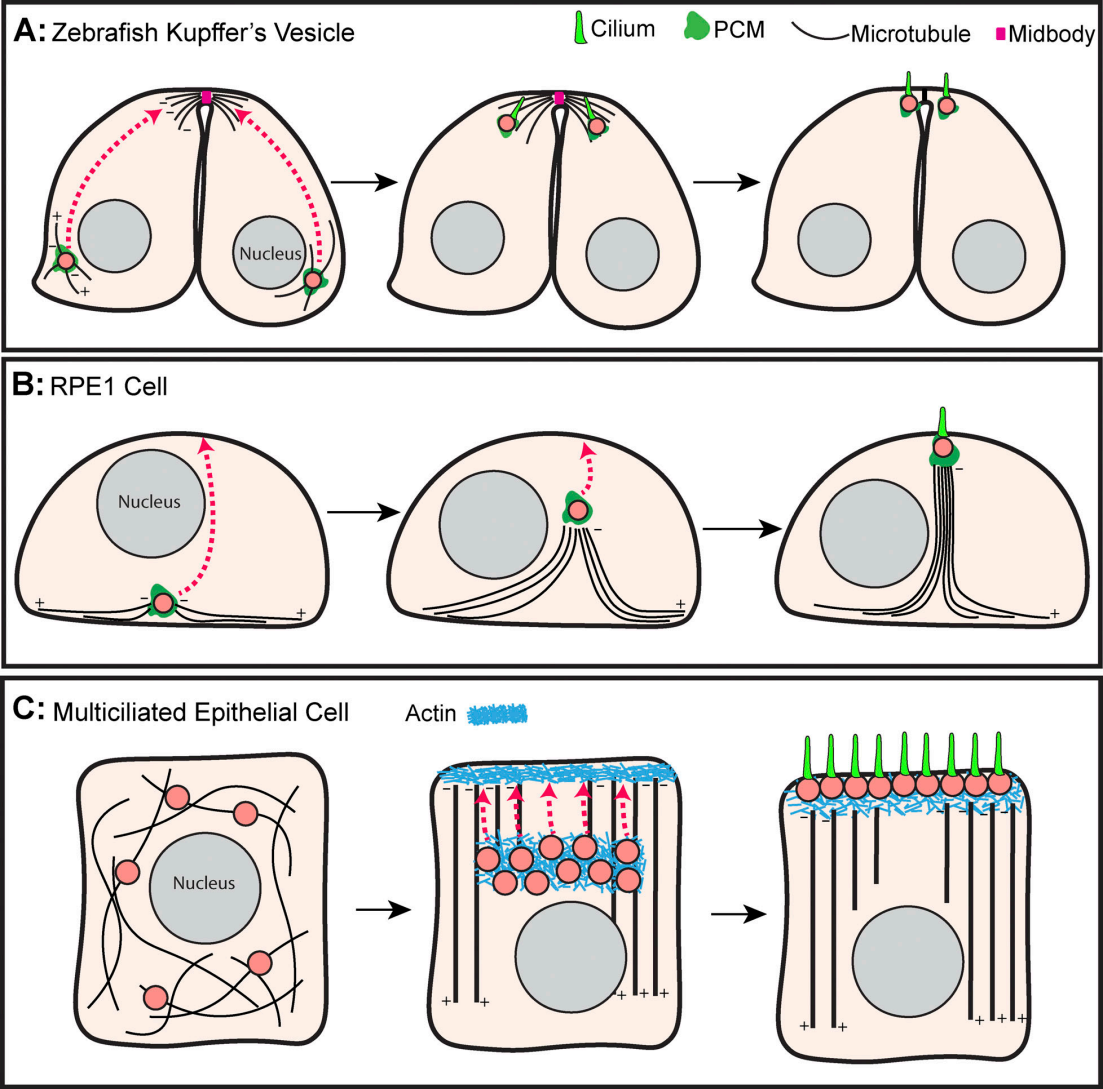
**Centrosome transport for ciliogenesis. (A)** During cytokinesis in the developing Kupffer’s vesicle, the centrosome (pink) migrates from the basal side of the cell to the cytokinetic furrow in a manner dependent upon the protein Pericentrin (magenta). The centrosomes associate with the midbody (pink) microtubules prior to abscission. After abscission, each centriole will form a single motile cilium. **(B)** In RPE-1 cells, the centrosome (pink) migrates from the basal to the apical surface to form a primary cilium. Migration occurs via the reorganization of the interphase microtubule array (black) pushing the centrosome through the cytoplasm. **(C)** In multiciliated cells, centrioles (pink) are amplified and then become enveloped in an actin matrix (light blue). The centrioles then migrate apically where the actin matrix forms the subapical actin network that anchors the centrioles prior to cilia (green) formation. +/− indicates microtubule polarity.

During fish morphogenesis, the left–right asymmetry is established by a transient spherical organ consisting of mono-ciliated cells known as the Kupffer’s vesicle (Essner et al., 2005). It was recently shown that before abscission, the centrosome migrates through the cell to the luminal surface of the Kupffer’s vesicle in a manner dependent upon the Rab11 GTPase as well as the centrosomal protein Pericentrin (Fig. 3 A) (Krishnan et al., 2022). Interestingly, like the movement of centrosomes during cytokinesis in some cell types (Fig. 2 A), the centrosomes of the Kupffer’s vesicle are associated with the midbody microtubules near the luminal surface (Fig. 3 A).

Another system used to investigate centrosome migration for ciliogenesis is human RPE-1 cells. Upon spatial confinement, RPE-1 cells exit the cell cycle, and the normally basally localized centrosome migrates to the apical cell surface and forms a primary cilium. This migration is independent of apically localized microtubule pulling machinery as centrosome migration precedes apical Dynein accumulation (Pitaval et al., 2017). Instead, centrosome migration is driven by the rapid bundling and stabilization of the microtubule network which forms a column that pushes the centrosome toward the apical pole (Fig. 3 B) (Pitaval et al., 2017). Microtubule cytoskeleton remodeling is downstream of a RhoA-mediated increase in actomyosin contractility via Myosin II phosphorylation (Pitaval et al., 2010); however, the precise relationship between actomyosin contractility and microtubule bundling is yet to be determined.

These two examples demonstrate that cells use highly diverse mechanisms to position centrosomes for primary ciliogenesis. Future work will be required to further dissect the molecular machinery required to position centrosomes in these various contexts. Of particular interest is identifying the differences between centrosome positioning in cells in which the cilia are formed prior to docking at the plasma membrane (intracellular ciliogenesis pathway) and those in which the cilia are formed after (extracellular ciliogenesis pathway) (Sorokin, 1962, 1968; Shakya and Westlake, 2021). The intracellular ciliogenesis pathway involves the docking of vesicles to the distal end of the centriole where they fuse to form what’s called the ciliary vesicle (Lu et al., 2015; Ganga et al., 2021). Recent work has shown that inhibiting Rab11 and Rab35 GTPases did not prevent intracellular cilia formation but prevented centrosome movement to the apical cell membrane, raising the possibility that the vesicle trafficking machinery can directly contribute to centrosome motility (Aljiboury et al., 2023).

The formation of the ciliary vesicle at the distal tip of the centriole and the docking of centrioles to the plasma membrane requires centriolar structures called distal appendages (Mansour et al., 2021). Multiple genes coding for proteins that constitute the distal appendages have been linked to ciliopathies including Joubert syndrome, infantile nephronitis, polydactyly, and more (Adly et al., 2014; Failler et al., 2014; Wheway et al., 2015; Mansour et al., 2021). Importantly, although many of the mutations in these genes result in cells with fewer cilia, little characterization has been done on the positioning or motility of centrosomes prior to ciliogenesis.

### Centriole motility in multiciliated cells

In some epithelial tissues such as oviducts, airways, and the spinal canal, cells greatly expand the number of centrioles needed to nucleate multiple motile cilia (Lyu et al., 2024). These cilia function to move fluids such as mucus or cerebrospinal fluid through the lumen of the epithelia. Failure to correctly form motile cilia can result in a series of diseases termed motile ciliopathies such as hydrocephalus, infertility, and chronic respiratory problems (Reiter and Leroux, 2017; Focşa et al., 2021)

Generating these large numbers of motile cilia requires massive amplification of centrioles, which then migrate to the apical surface of the epithelium where they become basal bodies that nucleate ciliary axonemes (Fig. 3 C). While much is known about the amplification of centrioles (Brooks and Wallingford, 2014; Spassky and Meunier, 2017), less is known about the mechanism by which this large population of centrioles migrates and docks to the apical surface.

Three decades ago, a series of studies using the ciliated quail oviduct investigated how multiple centrioles can migrate within the same cell for ciliogenesis. Using pharmacological perturbation and fixed cell analysis, it was shown that microtubules were not essential for centriole migration (Boisvieux-Ulrich et al., 1989a, 1989b). However, perturbing the actin network did prevent centriole migration, indicating an actin-dependent transport mechanism (Lemullois et al., 1988; Boisvieux-Ulrich et al., 1990). Interestingly, stabilization of microtubules with Taxol did inhibit centriole migration, suggesting that microtubule dynamics are somehow important for centriole motility in the oviduct (Boisvieux-Ulrich et al., 1989a, 1989b).

It was another decade before the use of model organisms such as *Xenopus*, zebrafish, and mice enabled the genetic dissection of the cytoskeletal regulators driving the migration and docking of centrioles at the plasma membrane. It became clear that the formation of a subapical actin meshwork downstream of the cytoskeletal regulators, Rac1, Ezrin, ELMO, and DOCK1 was essential for centriole positioning and ciliogenesis (Pan et al., 2007; Epting et al., 2015). The knockdown of these proteins in multiciliated *Xenopus* epidermal cells and zebrafish embryonic pronephric duct cells caused defective assembly of the apical actin network and resulted in the cytoplasmic positioning of centrioles.

Using fixed cell microscopy, it was not possible to distinguish between a failure in centriole migration and a failure in centriole docking, both of which would result in mispositioned centrioles. The advent of live cell imaging distinguished between these two possibilities, revealing that prior to migration, the centrioles were surrounded by a pool of actin (Fig. 3 C), and that additional actin assemblies were positioned at the apical cell surface. Centrioles then migrated apically en masse, appearing to use the pool of actin for centriole docking (Ioannou et al., 2013). Knockdown of the nucleotide-binding protein Nubp1 resulted in disorganization of this internal actin meshwork and a failure in centriole migration. While this suggests that actin is required for centriole migration to the cortex, it does not rule out an additional role for actin in centriole-cortical docking.

The above experiments led to the conclusion that actin, not microtubules, drove centriole migration to the apical cortex in multiciliated cells. However, this appears to be context-dependent as the vertebrate multiciliated olfactory sensory neurons do use microtubules to move centrioles (Ching et al., 2022). One possible general explanation for relying on microtubules rather than actin in neurons is the long distance traveled by the centrioles. The olfactory neuron extends a 50–100 μm dendrite toward the apical surface of the olfactory epithelium, a much longer distance than migration within epithelial cells.

In conclusion, centrioles that form the basal bodies of both motile and non-motile cilia must migrate from the cytoplasm to the cell cortex. The mechanisms seem to diverge between the requirement of the microtubule cytoskeleton and the actin cytoskeleton. However, the use of actin and microtubules might not be mutually exclusive. For example, although an intracellular actin meshwork surrounding the motile centrioles was identified, a dense microtubule network was also observed between the centrioles and the cell cortex (Fig. 3 C) (Ioannou et al., 2013). Thus, one possibility is that the actin meshwork clusters centrioles, while the microtubule network pulls the centrioles to the cell cortex (Laan et al., 2012). This model explains how Taxol treatment inhibited centriole motility in epithelia (Boisvieux-Ulrich et al., 1989a, 1989b; Ching et al., 2022). Indeed, fixed experiments performed on mouse ependymal cells have shown that prior to migration, centrioles are closely associated with the microtubule network and that after migration, the microtubule network is highly polarized in an apical to basal direction, meaning that the microtubule network is remodeled coincident with the repositioning of centrosomes (Al Jord et al., 2017). This model would contrast with evidence that colchicine and nocodazole do not block centriole migration (Boisvieux-Ulrich et al., 1990), although it is worth noting that colchicine is ineffective against stabilized microtubules, and that primary ciliogenesis in RPE-1 cells was only sensitive to colchicine treatment at specific stages of the ciliogenesis process, despite being microtubule dependent (Pitaval et al., 2017). Thus, it is important to revisit the effects of microtubule depolymerizing agents and therefore the role of the microtubule cytoskeleton on centrosome positioning in multiciliated epithelial cells.

### Centriole motility in asymmetric cell division

The neural stem cells (neuroblasts) of *D. melanogaster* divide consistently along the same orientation axis, which is in perfect alignment with the axis of polarity. The alignment of these axes is important for reliable segregation of cell fate determinants, allowing these cells to self-renew while also generating the cells required for the formation of the central nervous system (Loyer and Januschke, 2020). Importantly, neuroblast asymmetric division relies on proper centriole motility, the mechanism of which was only recently revealed.

**Figure 4.**
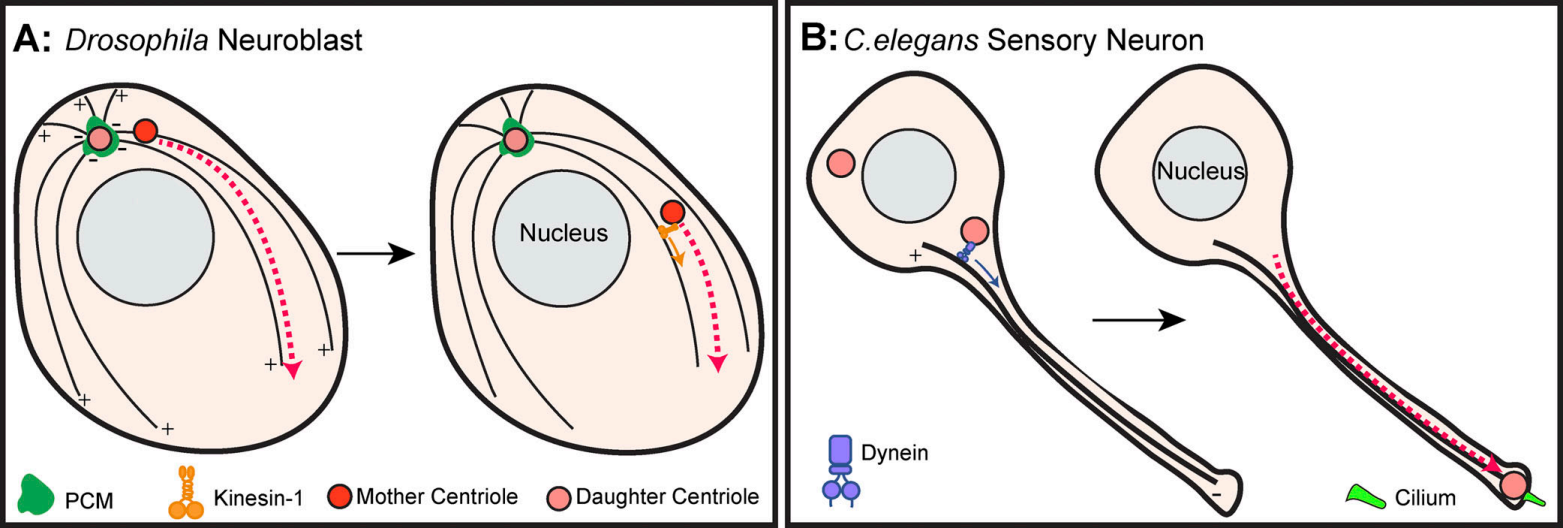
**Direct centriole transport in invertebrates. (A)** In the early interphase, *Drosophila* neuroblasts establish asymmetric pericentriolar material (dark green) distribution around the two centrioles (red and pink). The mother centriole (red) interacts with Kinesin-1 (orange) which then moves it in a plus end–directed manner along the microtubule network. As the cell approaches prophase, the centriole stops moving on the microtubule network and recruits pericentriolar material for bipolar spindle formation (not illustrated here). **(B)** After an extension of the *C. elegans*’ sensory neuronal dendrite, one of the two neuron centrioles (pink) migrates along the microtubule network from the cell body to the distal tip of the axon where it forms the sensory cilium (green). The centriole migrates via an interaction with the minus end–directed microtubule motor Dynein (purple). +/− indicates microtubule polarity.

Neuroblasts ensure their robust asymmetric cell division through a well-documented atypical centrosome cycle. Here, one centriole (the mother) sheds its PCM at the mitotic exit, while the daughter centriole maintains PCM and forms an apical MTOC that maintains the polarity of these cells throughout the interphase (Rusan and Peifer, 2007; Rebollo et al., 2007; Januschke and Gonzalez, 2010). The mother centriole then becomes highly mobile, migrating from the apical to the basal side of the interphase cell (Fig. 4 A). Recent work revealed that the mother centriole moves on the microtubules nucleated by the apical daughter centriole, using them as tracks to travel from the apical to the basal side of the interphase neuroblast (Hannaford et al., 2022). This plus-end directed microtubule transport is dependent upon the motor protein Kinesin-1, which is activated by its interactor MAP7 to trigger processive movement (Gallaud et al., 2014; Métivier et al., 2019). Kinesin-1 interacts with *Drosophila* Pericentrin-like-protein (PLP) on the surface of the centriole, which is necessary for mother centriole motility (Lerit and Rusan, 2013; Hannaford et al., 2022). This newly discovered mechanism is important as the apical to basal centriole motility is required for proper spindle orientation regulation and stem cell fate (Lerit and Rusan, 2013). Future work will be required to test if Kinesin-1 plays the same function in other contexts, such as the long-distance centriole migration in olfactory sensory neurons mentioned above.

The age-dependent segregation of centrosomes is not specific to *Drosophila* stem cells but has been observed in yeast, mice, chickens, and humans (Vallen et al., 1992; Wang et al., 2009; Tozer et al., 2017; Royall et al., 2023). In the budding yeast *Saccharomyces cerevisiae*, the spindle pole body (SPB; centrosome equivalent) replicates once per cell cycle (Kilmartin, 2014), and the older (mother) SPB is segregated preferentially into the bud. Proper positioning of the mother SPB toward and into the bud relies on SPB-microtubule interaction with cortical motor proteins positioned near the bud neck. This mechanism drives the older SPB into the bud while the younger SPB remains in the mother cell. Therefore, in contrast to the *Drosophila* neuroblast, the age-dependent segregation of the SPB is a form of indirect motor transport (Pereira et al., 2001).

In vertebrate neural stem cells, the mother centrosome is retained by the stem cell. The knockdown of the microtubule anchoring protein Ninein caused the random segregation of centrosomes in both mouse and human neural progenitors causing the loss of stem cells and precocious neuronal differentiation (Royall et al., 2023; Wang et al., 2009). It is not clear how Ninein promotes the retention of the older centrosome by the neural progenitors; however, Ninein specifically localizes to mother centrosomes (known to nucleate and anchor stable microtubules) where it recruits other microtubule regulators such as Dynactin, KIF2A, and CEP170 (Gasic et al., 2015; Mazo et al., 2016). These functionally asymmetric centrosomes could lead to the preferential anchoring of the mother centrosome at the ventricular surface of the developing cortex, leading to its preferential segregation into the stem cell upon asymmetric cell division. Importantly, the dynamics of the daughter centrosome in G2 have not been investigated, and it will be interesting to see whether, like the *Drosophila* neural stem cells, its positioning contributes to asymmetric cell division.

### Centriole motility in neurons

Motile centrioles have also been observed in the sensory neurons of *C. elegans* (Heiman and Shaham, 2009). Unlike the vertebrate olfactory neurons, *C. elegans*’ sensory neurons are monociliated. To form the sensory cilium, one centriole migrates from the cell body to the dendritic tip where it is converted into a basal body and nucleates the cilium (Fig. 4 B). Like the migration of centrioles observed during *Drosophila* neuroblast asymmetric cell division, only one of the two centrioles is migratory, though it is not known if this is the mother or the daughter centriole. Analysis of microtubule dynamics revealed that most microtubules were nucleated from the dendritic tip toward the cell body. Therefore, unlike *Drosophila* neuroblasts, the centriole migrates in a minus end–directed manner (Li et al., 2017). This study found (1) that Dynein light chain colocalized with the centriole during the dendritic migration, (2) that knockdown of components of the Dynein complex perturbed centriole migration, and (3) that the Dynein light chain interacted with the centriole protein Sas-5. All these data suggest that the centrioles are transported along the microtubule network (Li et al., 2017). Interestingly, in *D. melanogaster* sensory neurons, loss of Pericentrin-like protein also disrupted the positioning of centrioles in the sensory dendrites (Galletta et al., 2014). Thus, a conserved mechanism of Dynein transport of centrioles in neurons is quite plausible but would require additional future investigation to separate the potential role of Dynein pulling forces with direct transport. Pericentrin, for example, is a known interactor of Dynein and localizes to centrioles in mammalian sensory neurons (Mühlhans et al., 2011; Purohit et al., 1999), though its function in centriole positioning has not been tested.

### Positioning centrioles during gametogenesis

**Figure 5.**
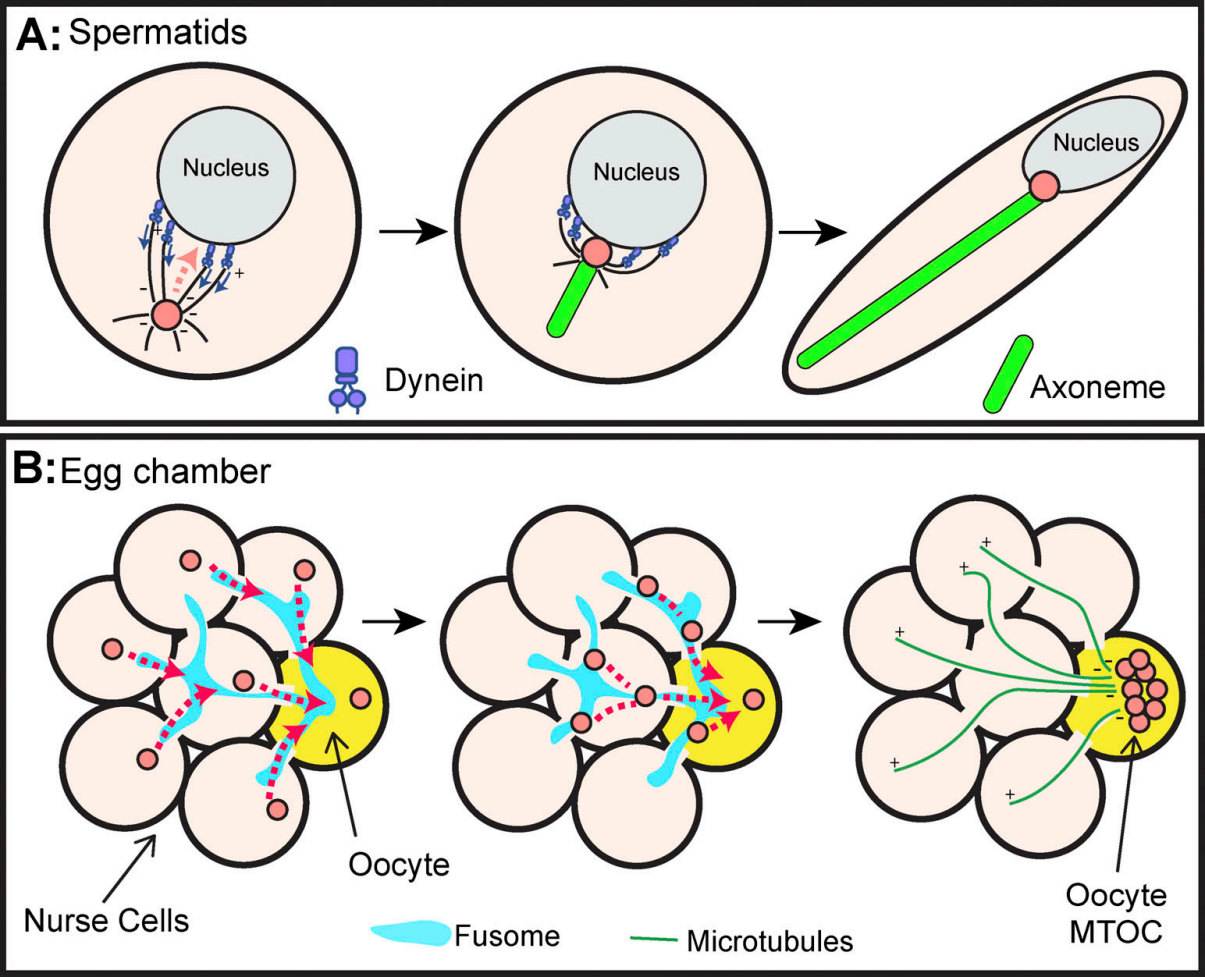
**Centrosome positioning in gametogenesis. (A)** During spermiogenesis, the centrosome (pink) acts as a linker between the nucleus (head) and axoneme (tail). To facilitate this interaction, dynein (purple) accumulates on one side of the nuclear membrane. Dynein then pulls on centrosomal microtubules to position the centrosome adjacent to the nucleus where it then builds the sperm axoneme (green). **(B)** Oogenesis occurs in a cyst of interconnected cells. To ensure only one MTOC is present in the oocyte (yellow), centrosomes (pink) migrate through the nurse cells (nude) along the fusome (blue). +/− indicates microtubule polarity.

In many metazoan organisms, the process of centrosome motility plays a critical role during spermatogenesis and oogenesis. In males, centrosomes must be positioned near the haploid sperm head to ensure proper attachment of the sperm head and tail (Fig. 5 A). In females, centrosomes must migrate through the cyst to the oocyte to form a single microtubule organizing center, which is essential for the maternal contribution of proteins and RNA (Fig. 5 B) (Mahowald and Strassheim, 1970; Nashchekin et al., 2021).

During sperm development, the centrosome will migrate toward the spermatid nucleus (head) and form a stable attachment before extending the axoneme from its distal end (Chemes and Rawe, 2010). Failure to migrate to the surface of the nucleus results in what are known as headless or acephalic sperm, which are incapable of fertilization (Yuan et al., 2015). The structure of sperm is well conserved, and genetic studies in *Drosophila* have unveiled a working model for how the migration of the centrosome to the nuclear envelope is coordinated. In this model, Dynein localizes to the nuclear envelope, and over time, becomes polarized to one side of the nucleus (Anderson et al., 2009; Sitaram et al., 2012). This polarization is likely critical for positioning the centrosome as disruption of the Dynein complex correlated with loss of the centriole–nucleus connection (Kracklauer et al., 2010; Wu et al., 2016). Live imaging of cells, as they exited meiosis, revealed that the microtubules emanating from the centrosome contacted the nucleus, thereby reeling the centrosome to the nuclear surface (Galletta et al., 2020).

The mechanism of centrosome movement into the oocyte has predominantly been studied in the egg chambers of *D. melanogaster*. During early oogenesis, a 16-cell cyst is formed, where 15 cells are designated nurse cells that supply maternally contributed protein and RNA to the designated oocyte via microtubule-transport that traverses ring canals (cytoplasmic bridges formed through incomplete mitoses [Fig. 5 B]). Electron microscopy studies of early *Drosophila* oogenesis described centrosomes located in all 16 cyst cells; however, at later stages, centrosomes had migrated through the ring canals and clustered in the oocyte (Mahowald and Strassheim, 1970). Importantly, immunofluorescence confirmed these findings and demonstrated that the centrosomes appear to localize to a microtubule-rich structure known as the fusome (Fig. 5 B) (Grieder et al., 2000). Centrosome migration to the oocyte was highly dependent on the microtubule motor Dynein that moves along the polarized microtubule network from the anterior to the posterior end of the cyst (Bolívar et al., 2001). It is unclear whether the centrosomes migrate via direct transport like the *C. elegans* sensory neuron and *Drosophila* neuroblast, or whether they move via indirect transport via centrosomal microtubules. Importantly, these centrosomes appear functionally intermediate between a centriole and a centrosome in terms of its microtubule organizing capacity as they contain little PCM and few microtubules (Bolívar et al., 2001; Grieder et al., 2000).

The formation of oocytes from cysts of germ cells has been observed not only in flies but also frogs, fish, and mice (Spradling et al., 2022), and the migration of centrosomes through the oocyte appears to be conserved. In the mouse germline, by embryonic day 10, primordial germ cells have migrated to the gonad and formed an interconnected cyst of up to 30 cells. Analysis of germ cells revealed that on embryonic day 14.5, most cells contained single centrosomes; however, by E17.5 some cells contained multiple, suggesting that they were transported through the cyst into the oocytes (Lei and Spradling, 2016). Like *Drosophila*, these centrosomes converge to form a single microtubule organizing center that facilitates the transport of material necessary for oocyte fate and polarity (Lei and Spradling, 2016; Niu and Spradling, 2022).

Investigating the mechanisms of centrosome positioning in gametes demonstrates the difference between the short- and long-range transport of centrosomes. In oogenesis, the centrosomes must undergo a long-distance migration through the developing germline cyst. These centrosomes do not have expanded PCM; however, it is unclear whether the motility is direct or indirect via residual microtubule nucleating capability. Meanwhile, in spermiogenesis, the centrosome has an expanded PCM that promotes indirect migration via centrosomal microtubules and nuclear envelope-anchored Dynein; importantly, the PCM in spermatocytes is restricted to the proximal end of the centriole, which facilitates the perpendicular attachment of the centriole to the nucleus (Galletta et al., 2020). The mechanism of sperm centrosome motility is therefore better understood; however, it is not known what triggers the capture of centrosomal microtubules as the cell exits meiosis. Understanding centrosome migration in the germline may therefore expand our knowledge of both male and female reproductive disease.

## Conclusion and perspective

Over 100 years ago, Theodor Boveri appreciated the importance of positioning the centrosome for the organization of cellular components and the proper segregation of these components upon cell division (Scheer, 2014). We now know that the centrosome does not simply mark the geometrical center of the cell, but the precise positioning of mature centrosomes, as well as immature centrioles, is a core feature of cellular polarization and organization throughout the eukaryotic lineage.

Centrosome positioning is well studied in the context of mature centrosome separation at the onset of mitosis. The separation of mitotic centrosomes is a form of indirect motor transport that requires motor proteins found in the nuclear envelope, at the cell cortex, and between antiparallel microtubules (Prosser and Pelletier, 2017; Tanenbaum and Medema, 2010; Valdez et al., 2023). This mitotic centrosome positioning is fundamentally important for proper chromosome segregation, and multiple attempts to generate chemotherapeutic agents have targeted this process (Box 2). However, these attempts have largely been unsuccessful, and an understanding of how cancer cells adapt to and tolerate inhibitors targeting centrosome positioning is critical to identify further mechanisms regulating this process that can potentially be targeted for chemotherapy.

The positioning of centrosomes via indirect motor transport is not unique to mitosis. Spermiogenesis and immunological synapse formation require the capture of centrosomal microtubules by the motor protein Dynein followed by the shrinkage of the microtubules, resulting in centrosome translocation (Sitaram et al., 2012; Yi et al., 2013). However, this does not explain all indirect microtubule movements. For example, the drastic reorganization of the microtubule network that leads to centrosome migration to the apical cortex of RPE-1 cells for primary ciliogenesis occurs before Dynein concentrates at the apical pole (Pitaval et al., 2017). Thus, additional force-generating mechanisms must be involved, likely through motor proteins specific to each cellular context.

In other cell types, the migration of immature centrosomes/centrioles does not require the nucleation of microtubules by the centrosome. For example, in mammalian cells undergoing cytokinesis, *C. elegans’* sensory neurons and *D. melanogaster’s* neural stem cells, centrioles have been observed to move along the microtubule network, via direct motor transport. In the latter two examples, direct interaction between centriole components and the motor proteins Dynein or Kinesin-1 is required for centriole movement (Hannaford et al., 2022; Li et al., 2017).

Finally, in multiciliated cells, the centrioles must migrate to the apical surface of the cell in an actin-dependent manner. Live imaging revealed an actin meshwork surrounding the centrioles, but it is unclear how this actin network propels the centrioles to the apical surface (Ioannou et al., 2013). Interestingly, the microtubule network also becomes highly polarized in multiciliated cells, and prior to migration, the centrioles are spaced out along the microtubule network (Al Jord et al., 2017). It is therefore possible that direct motor transport facilitates the apical migration of multiple centrioles.

A common theme of both centrosome and centriole motility is the coordination of microtubule motors. Indirect motor transport requires the localization and activation of Dynein (and likely other unknown motors) to specific cellular compartments. Direct motor transport requires the interaction of motor proteins with the centriole/centrosome and the careful regulation of these motors to achieve directionality. We are only now beginning to uncover the diverse mechanisms that regulate the direct motor transport of centrioles, which will require combining live cell imaging, in vitro reconstitution, and careful genetic ablation. We are at the early stages of understanding the complex regulation of motor-based centriole movements, and it will be important to identify all the molecular motors involved and their adaptors.

Critically, it is essential that new experimental tools and biophysical models are utilized that allow for the specific modulation of the centrosome and centriole positioning machinery. Adhesive micropatterning allows researchers to control cell shape, adhesion, and spindle orientation (Théry, 2010; Fink et al., 2011). At its simplest, this allows the standardization of these parameters to prevent artifacts affecting experimental results; however, micropatterning has been effectively used to investigate how modulation of these parameters affects the positioning of centrosomes in the cell (Jimenez et al., 2021; Pitaval et al., 2010, 2017; Lima et al., 2024).

The generation of biophysical models in silico enables scientists to manipulate individual molecular populations and rapidly test hypotheses free from experimental limitations. Models have already been used to interrogate the mechanisms of centrosome movement during prophase centrosome separation, immune synapse formation, as well as centrosome centering (Som et al., 2019; Gros et al., 2021; Mercadante et al., 2021; Wu et al., 2023). These models allow the precise manipulation of specific protein populations, for example, modulating the clustering of Dynein or inactivating Dynein at specific locations within the cell.

The advent of live cell imaging has revolutionized our understanding of how centrosomes and centrioles move in cells; however, the inability to precisely modulate protein function with high spatiotemporal resolution was prohibitive to mechanistic understanding. Modern optogenetic approaches allow the light-sensitive inhibition of microtubule motors and cell contractility as well as the time-sensitive clustering and therefore, inactivation of any protein of interest (Nijenhuis et al., 2020; Nagpal et al., 2023, *Preprint*; Bugaj et al., 2013; Aljiboury et al., 2023). A combination of these approaches in diverse cell and tissue types will usher in a new and deep understanding of the direct and indirect mechanisms regulating centrosome and centriole positioning.
